# Supplementary material for: Time diffraction-free transverse orbital angular momentum beams
Source: Nat Commun. 2022 Jul 11;13:4021. doi: 10.1038/s41467-022-31623-7 (PMC9276663; doi:10.1038/s41467-022-31623-7)
Supplement: Supplementary file 1 — Supplementary Information [file 41467_2022_31623_MOESM1_ESM.pdf]

Supplementary information:
Time diffraction-free transverse orbital angular momentum beams

Wei Chen [1,#,*], Wang Zhang [1,#], Yuan Liu [1], Fan-Chao Meng [2,3], John M. Dudley [2], Yan-Qing Lu [1,*]

[1]National Laboratory of Solid State Microstructures, Key Laboratory of Intelligent Optical Sensing and Manipulation, College of Engineering and Applied Sciences, and Collaborative Innovation Center of Advanced Microstructures, Nanjing University, Nanjing 210093, China

[2]Institut FEMTO-ST, Université Bourgogne Franche-Comté CNRS UMR 6174, Besançon, 25000, France

[3]State Key Laboratory of Integrated Optoelectronics, College of Electronic Science and Engineering, Jilin University, 2699 Qianjin Street, Changchun 130012, China

[#]These authors contributed equally: Wei Chen, Wang Zhang.

[*]Correspondence to: wchen@nju.edu.cn, yqlu@nju.edu.cn

Supplementary Note 1

Spatiotemporal Bessel (STB) vortices

Let us consider a polychromatic wave packet which is an impulsive ring with a spiral phase on the space frequency (k_x)–time frequency (ω) plane, *i.e.*,

$$\tilde{E}(k_x,\Omega)=\tilde{E}_l(\kappa,\varphi)=\delta(\kappa-R_0)e^{il\varphi}, \tag{1}$$

where $\Omega=\gamma(\omega-\omega_0)$ relates to the detuning frequency, ω_0 is the central frequency, $\gamma=\Delta k_x/\Delta\omega$ is the reduction coefficient for temporal and spatial scale consistency, $\kappa=\sqrt{k_x^2+\Omega^2}$ and $\varphi=\tan^{-1}(k_x/\Omega)$ are the polar coordinates on the k_x–ω plane, and R_0 is the modulated radius. The two-dimensional Fourier transform of Eq. (1) yields the field on the space (x)–time (τ) plane, that is,

$$E_l(x,\tau)=F\cdot T\cdot\{\tilde{E}_l(k_x,\Omega)\}=\frac{1}{2\pi}\int_{-\infty}^{\infty}\int_{-\infty}^{\infty}\tilde{E}_l(k_x,\Omega)e^{i\Omega\tau}e^{ik_x}dk_x d\Omega. \tag{2}$$

After a polar coordinate transformation, Eq. (2) becomes

$$\begin{aligned}E_l(\rho,\theta)&=F\cdot T\cdot\{\tilde{E}_l(\kappa,\varphi)\}\\&=\frac{1}{2\pi}\int_0^{2\pi}\int_0^{\infty}\tilde{E}_l(\kappa,\varphi)e^{i\kappa\rho\cos\varphi\cos\theta}e^{i\kappa\rho\sin\varphi\sin\theta}\kappa d\kappa d\varphi\\&=\frac{1}{2\pi}\int_0^{2\pi}\int_0^{\infty}\tilde{E}_l(\kappa,\varphi)e^{i\kappa\rho\cos(\varphi-\theta)}\kappa d\kappa d\varphi\end{aligned}, \tag{3}$$

where $\rho=\sqrt{x^2+\tau^2}$, $\theta=\tan^{-1}(x/\tau)$ and $\tau=t-z/v_g$ is the retarded time in the pulse frame where v_g is the group velocity. Using Eq. (1) and (3), along with the integral definition of Bessel functions[1], we have

$$\begin{aligned}E_l(\rho,\theta)&=\frac{1}{2\pi}\int_0^{2\pi}\int_0^{\infty}\delta(\kappa-R_0)e^{il\varphi}e^{i\kappa\rho\cos(\varphi-\theta)}\kappa d\kappa d\varphi\\&=(i)^l e^{il\theta}\int_0^{\infty}J_l(\kappa\rho)\delta(\kappa-R_0)\kappa d\kappa\\&=(i)^l R_0 e^{il\theta}J_l(R_0\rho)\end{aligned}, \tag{4}$$

where J_l is the l-order Bessel function of the first kind. Eq. (4) indicates that the fields with ST spectra described by Eq. (1) are strictly equivalent to the high-order STB vortices[2].

Supplementary Note 2

Inverse design of spiral phase

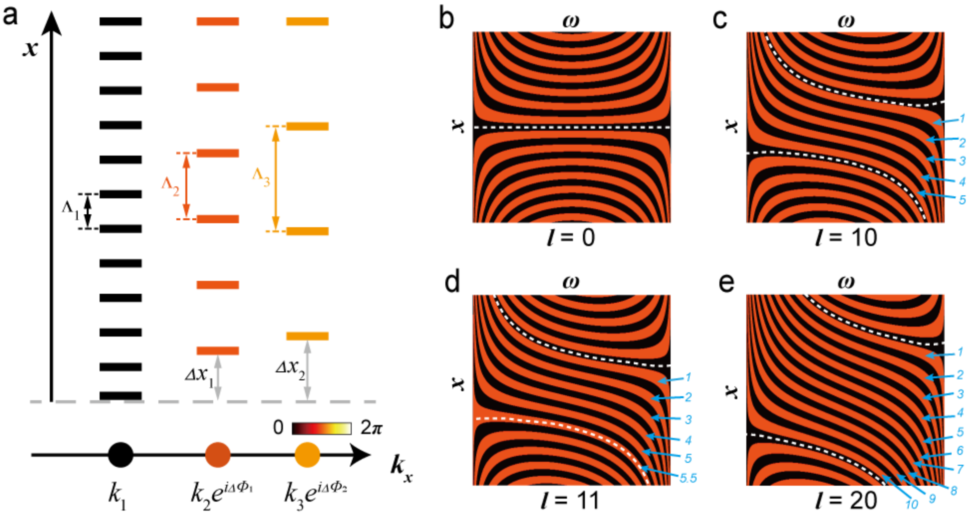

Supplementary Fig. 1 | Principle of inverse design of phase. a, Principle. **b–e**, Phase patterns for generating STB vortices with different topological charges of *l* = 0, 10, 11 and 20. The white dashed lines in **b–e** mark the positions of the left and right main lobes, and the blue arrows point out the accumulated dislocation of phase between the left and right main lobes.

Suppose a ST frequency point $\tilde{E}(\Omega, k_n) = |E_n|\delta(k_x - k_n)e^{i\phi_n}$ on the k_x–ω plane, where $|E_n|$ is the amplitude and ϕ_n is the phase. Its counterpart on the real *x* axis can be obtained by taking Fourier transform along the k_x axis, *i.e.*,

$$\begin{aligned} E(\Omega_n, x) &= F \cdot T \cdot \{\tilde{E}(\Omega_n, k_n)\} \\ &= \frac{1}{2\pi}\int_{-\infty}^{\infty} |E_n|\delta(k_x - k_n)e^{i\phi_n}e^{ik_x x}dk_x \\ &= \frac{1}{2\pi}|E_n|e^{ik_n\left(x-\frac{\phi_n}{k_n}\right)} \end{aligned} \quad (5)$$

One can see that such frequency point is actually a location-shifted grating, where the corresponding period and displacement is $\Lambda_n = 2\pi/k_n$ and $\Delta x_n = \phi_n/k_n$, respectively. By further binarizing this grating, a pair of conjugate frequency points $k = \pm k_n$ can be obtained, whose phase satisfies $\phi_n = -\phi_{-n}$. In particular, for a STB vortex, its ST spectrum is described by Eq. (1). Hence, according to Eqs. (1) and (5), we can obtain the *x*–*ω* phase of a STB vortex that possesses an impulse ring modulation with a spiral phase on the k_x–ω plane. For each STB vortex, the amount of dislocation between the left and right main lobes in the *x*–*ω* phase diagram reflects its topological charge, *i.e.*, the dislocation of phase between the left and right main lobes can be described as $(l/2) \times \pi$. As shown in Supplementary Figs. 1b–e, for STB vortices with topological

charges of l = 0, 10, 11, and 20, the corresponding dislocations are 0, 5π, 5.5π, and 10π. Notably, the phase binarization brings additional high-frequency components, which can be removed by spatial filtering in the experiment.

Supplementary Note 3

Theoretical analysis of the equivalence of the proposed *x*–*ω* modulation scheme

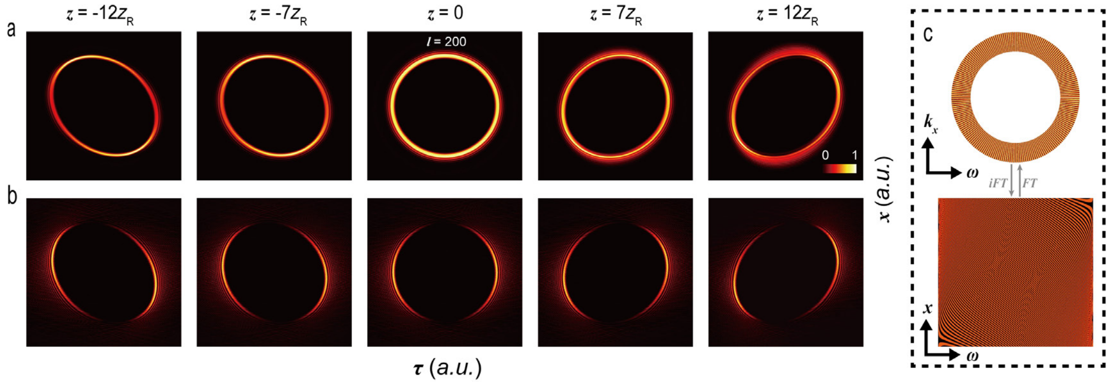

Supplementary Fig. 2 | Simulated results of propagation dynamics of two STB vortices with the same topological charge of *l* = 200 but undergoing different ST spectra modulation. a, Simulated intensities through immediate k_x–ω modulation. **b**, Simulated intensities through proposed x–ω modulation. **c**, Relationship between immediate k_x–ω and proposed x–ω modulation. The positions are marked at the top of each column (of **a** and **b**), where Z_R is the Rayleigh distance of a Gaussian beam with the same full-width at half-maximum of the corresponding first-order STB vortex.

As shown in Supplementary Fig. 2a, the ideal STB vortex, represented as a perfect impulse ring with a spiral phase on the k_x–ω plane, has a uniform mode distribution of energy. However, as we analysed in the main text, it is difficult to directly perform such immediate k_x–ω modulation in experiments because of the time-delayed spatial Fourier transform (SFT). Fortunately, regardless of the slight loss of energy in the *x* direction away from the centre (owing to the pixel accuracy), the immediate x–ω modulation scheme proposed in this work can be an effective method to generate STB vortices, as shown in Supplementary Fig. 2b.

Supplementary Note 4

Phase reconstruction of STB vortices

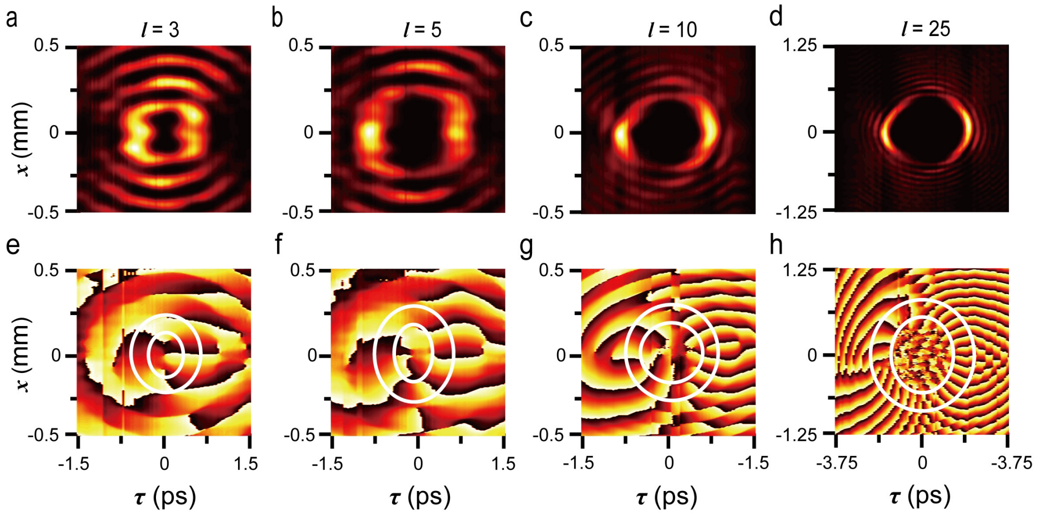

Supplementary Fig. 3 | Reconstructed intensities and phases of STB vortices with $l = 3$, 5, 10, and 25. a–b, Reconstructed intensities. **e–h**, Reconstructed phases accordingly. The white circles in **e–h** mark the spiral phases of each STB vortex. The corresponding topological charges are marked at the top of each column.

The space–time intensities of STB vortices with $l = 3$, 5, 10, and 25 are shown in Supplementary Figs. 3a–d. As shown in Supplementary Figs. 3e–h, the corresponding reconstructed phases verify the spiral phase of corresponding topological charges despite the slight distortion, implying the carried transverse OAM. Notably, for $l = 3$ and 5, the spatial bandwidth and temporal bandwidth of these STB vortices are set to Δk_x = ~61.5 rad/mm and $\Delta\lambda$ = ~6 nm, respectively. The measured similar widths of the STB vortices on the x–t plane (with $l = 5$ and 10) indicates that the widths of the STB vortices on the x and t axes are inversely proportional to the spatial and temporal spectral bandwidths, respectively.

Supplementary Note 5

Derivation of formula for the intrinsic dispersion factor $\boldsymbol{\beta_2^{\text{int}}}$

Using ST spectra of STB vortices described in Eq. (1) and general light-cone equation $k_x^2 + k_z^2 = (\omega/c)^2$ we can obtain the relation between the transmission constant k_z and the time frequency ω, that is,

$$k_z = \sqrt{(1/c^2 + \gamma^2)\omega^2 - 2\gamma^2\omega\omega_0 + \gamma^2\omega_0^2 - R_0^2}. \tag{6}$$

By setting $a = 1/c^2 + \gamma^2$ and $b = \gamma^2\omega_0^2 - R_0^2$, we have $k_z = \sqrt{a\omega^2 - 2\gamma^2\omega\omega_0 + b}$. Formally, we defined the intrinsic *n*-order dispersion factor[3]

$$\beta_n^{\text{int}} = \partial^n k_z(\omega)/\partial\omega^n|_{\omega=\omega_0}. \tag{7}$$

In this manner, we can get the second-order dispersion factor, *i.e.*,

$$\begin{aligned}
\beta_2^{\text{int}} &= \partial^2 k_z(\omega)/\partial\omega^2|_{\omega=\omega_0} \\
&= \partial^2\left(\sqrt{a\omega^2 - 2\gamma^2\omega\omega_0 + b}\right)/\partial\omega^2|_{\omega=\omega_0} \\
&= [ab - \gamma^4\omega_0^2]/\left(a\omega^2 - 2\gamma^2\omega\omega_0 + b\right)^{\frac{3}{2}}\Big|_{\omega=\omega_0}. \\
&= \left[\gamma^2\left(\frac{\omega_0^2}{c^2} - R_0^2\right) - \frac{R_0^2}{c^2}\right]/\left(\frac{\omega_0^2}{c^2} - R_0^2\right)^{\frac{3}{2}}
\end{aligned} \tag{8}$$

As discussed in the main text, the time diffraction of STB vortices can be perfectly predicted by the group dispersion delay model using the specific β_2^{int} calculated by Eq. (8).

Supplementary Note 6

Suppression of time diffraction by media dispersion engineering

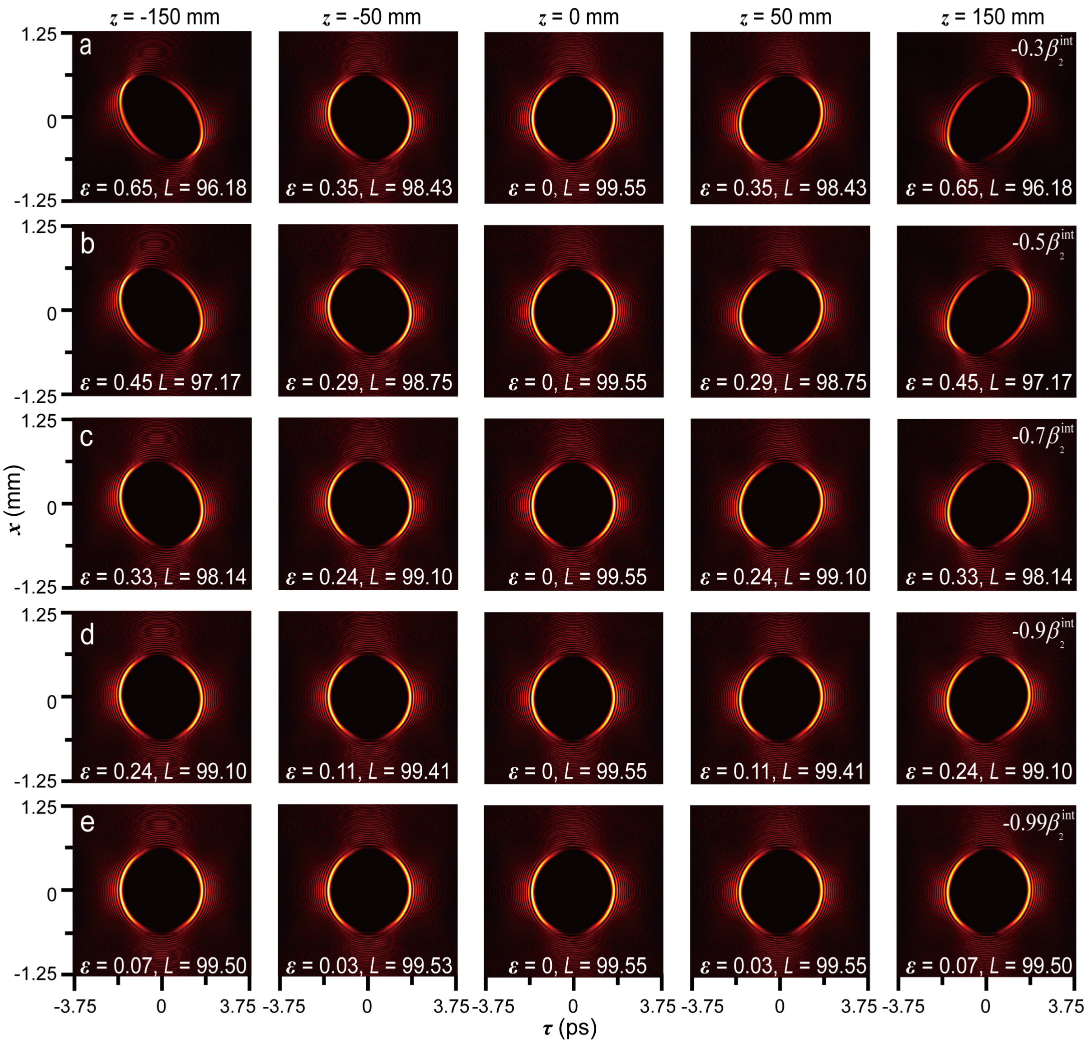

Supplementary Fig. 4 | Evolutions of an STB vortex with l = 100 propagating in virtual media with different negative dispersions. a–e, Simulated results with negative dispersions from $-0.3\beta_2^{\text{int}}$ to $-0.99\beta_2^{\text{int}}$. The corresponding media negative dispersion is plotted in top right corner of each row. The calculated eccentricities and integral OAM values are shown at the bottom of each panel.

Supplementary Note 7

Propagation dynamics of STB vortices with a lower *l*

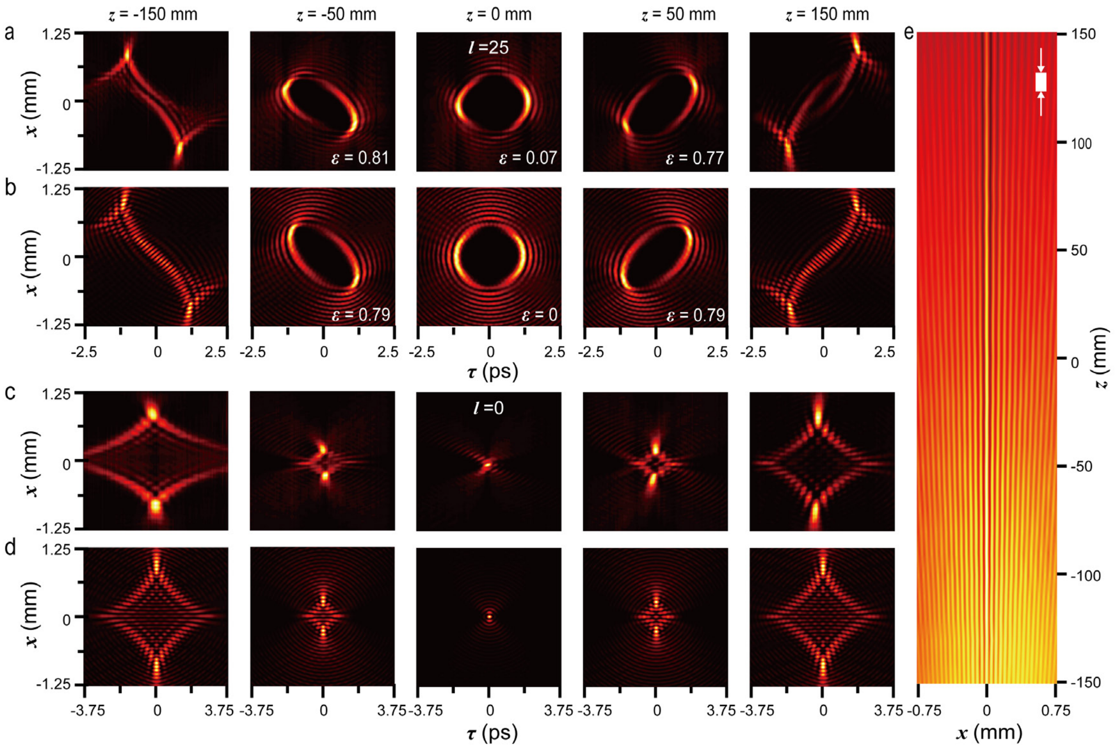

Supplementary Fig. 5 | Experimental and simulated results of propagation dynamics of two STB vortices with topological charges of *l* = 25 and 0. a, Measured intensities of an STB vortex with the topological charge of *l* = 25 at *z* = -150, -50, 0, 50, and 150 mm, where the position of the standard mode (described in Eq. (2)) is marked as *z* = 0 mm. **b**, Corresponding simulated results of **a**. **c**, Same as **a**, but with the topological charge of *l* = 0. **d**, Corresponding simulated results of **c**. The positions are marked at the top of each column in **a–d**. **e**, Measured time-averaged intensities of an STB vortex with *l* = 0 along *z*, where the solid white rectangle in the top-right corner represents the Rayleigh distance Z_R = 8.6 mm for the Gaussian beam with Δx = ~55 μm. The time-averaged intensities at different positions also reflect the slight divergence of the light beam in the experiment.

Supplementary Discussion

Impact of spectral uncertainty on time diffraction

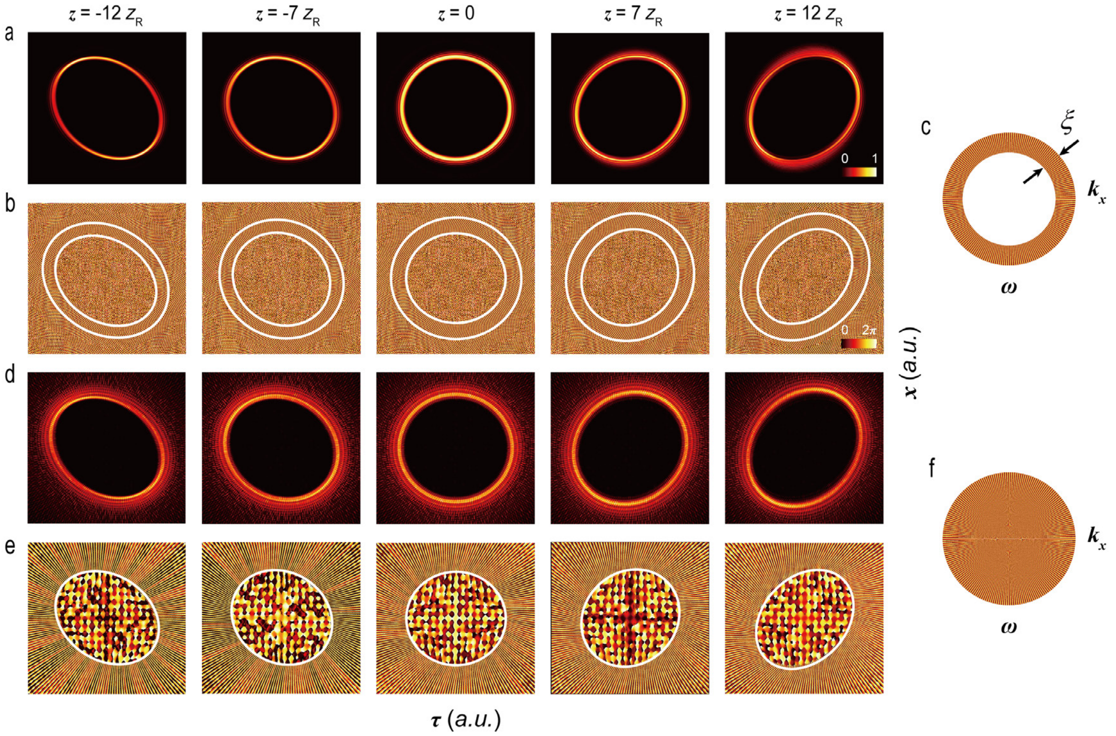

Supplementary Fig. 6 | Simulated results of propagation dynamics of an STB vortex and a Gaussian-like ST vortex with the same topological charge of *l* = 200 but different ST spectra. **a**, Simulated intensities of an STB vortex with low spectral uncertainty ξ, where the position of the standard mode is marked as $z = 0$. **b**, Corresponding phases of **a**. **c**, Corresponding ST spectra of **a**. **d–f**, same as **a–c** but for a Gaussian-like ST vortex with high spectral uncertainty. The white dotted circles in **b** and **e** mark the spiral phases of each ST beam. The positions are marked at the top of each column in **a**, **b**, **d**, and **e**, where Z_R is the Rayleigh distance of a Gaussian beam with the same full-width at half-maximum of the corresponding 0-order STB beam.

For an ideal STB vortex as described by Eq. (1), its ST spectrum is an impulse ring and the spectral uncertainty ξ is zero. Actually, such a spectral uncertainty cannot be achieved in practical experiments due to the limited precision of the devices (*e.g.*, the limited pixel and liquid crystal fill rate of the SLM). Nevertheless, here we show that the spectral uncertainty does not affect the intrinsic dispersion parameters. Considering the near-paraxial ($\Delta k_x \ll k_0$) and the quasi-monochromatic ($\Delta\omega \ll \omega_0$) condition, Eq. (8) can be further simplified, *i.e.*,

$$\begin{aligned}\beta_2^{\text{int}}(\omega_0) &= \left[\gamma^2\left(\frac{\omega_0^2}{c^2} - R_0^2\right) - \frac{R_0^2}{c^2}\right] / \left(\frac{\omega_0^2}{c^2} - R_0^2\right)^{\frac{3}{2}} \\ &\approx \left[\gamma^2\left(\frac{\omega_0^2}{c^2} - R_0^2\right)\right] / \left(\frac{\omega_0^2}{c^2} - R_0^2\right)^{\frac{3}{2}} \\ &\approx \gamma^2 \frac{c}{\omega_0}\end{aligned} \quad . \tag{9}$$

From Eq. (9), it can be clearly seen that intrinsic dispersion parameter β_2^{int} is independent to the R_0. This means that for a concentric ring with a central frequency ω_0, each ring experiences the same time diffraction during propagation. Therefore, the spectral uncertainty does not influence on the evolution of ST vortices (Supplementary Fig. 6).

Supplementary References

1 Oppenheim, A. V., Frisk, G. V. & Martinez, D. R. Computation of the Hankel transform using projections. *J. Acoust. Soc. Am.* **68**, 523–529 (1980).

2 Michael, D., Nathalie, M. & Michel, P. Spatiotemporal Bessel beams: theory and experiments. *Opt. Express* **17**, 18148–18164 (2009).

3 Agrawal, G. P. *Nonlinear Fiber Optics* 3rd edn (Academic, San Diego, 2001).
