# Supplementary material for: Time diffraction-free transverse orbital angular momentum beams
Source: Nat Commun. 2022 Jul 11;13:4021. doi: 10.1038/s41467-022-31623-7 (PMC9276663; doi:10.1038/s41467-022-31623-7)
Supplement: Supplementary file 3 — Description of Additional Supplementary Files [file 41467_2022_31623_MOESM3_ESM.pdf]

File name: Supplementary Movie 1

Description: Theoretical movie for propagation dynamics of an STB vortex with l = 200 generated by the immediate kx–ω modulation.

File name: Supplementary Movie 2

Description: Theoretical movie for propagation dynamics of the same STB vortex generated by the proposed x–ω modulation.

File name: Supplementary Movie 3

Description: Theoretical movie for propagation dynamics of an STB vortex with l = 1000 generated by the proposed x–ω modulation.

File name: Supplementary Movie 4

Description: Theoretical movie for propagation dynamics of an STB vortex with l = -1000 generated by the proposed x–ω modulation.
